# Neurocognitive mechanisms of emotional interference in native and foreign languages: evidence from proficient bilinguals

**DOI:** 10.3389/fnbeh.2024.1392005

**Published:** 2024-08-07

**Authors:** Nicola Del Maschio, Simone Sulpizio, Camilla Bellini, Gianpaolo Del Mauro, Matteo Giannachi, Duygu Buga, Davide Fedeli, Daniela Perani, Jubin Abutalebi

**Affiliations:** ^1^Centre for Neurolinguistics and Psycholinguistics, Faculty of Psychology, Vita-Salute San Raffaele University, Milan, Italy; ^2^Department of Psychology, University of Milano-Bicocca, Milan, Italy; ^3^Milan Center for Neuroscience (NeuroMI), University of Milano-Bicocca, Milan, Italy; ^4^Department of Diagnostic Radiology and Nuclear Medicine, University of Maryland School of Medicine, Baltimore, MD, United States; ^5^Research Department, VivaVoce Medical Center, Milan, Italy; ^6^Neuroradiology Unit, IRCCS Foundation Carlo Besta Neurological Institute, Milan, Italy; ^7^Nuclear Medicine Unit, San Raffaele Hospital, San Raffaele Scientific Institute, Milan, Italy; ^8^UiT The Arctic University of Norway, Tromsø, Norway

**Keywords:** bilingualism, Emotional Stroop, fMRI, cognitive control, emotion, implicit word processing

## Abstract

Currently available data show mixed results as to whether the processing of emotional information has the same characteristics in the native (L1) as in the second language (L2) of bilinguals. We conducted a functional magnetic resonance imaging (fMRI) experiment to shed light on the neurocognitive mechanisms underlying bilinguals’ emotional processing in L1 and L2 during an emotional interference task (i.e., the Emotional Stroop Task – EST). Our sample comprised proficient Italian-English bilinguals who learned their L2 during childhood mainly in instructional rather than immersive contexts. In spite of no detectable behavioural effects, we found stronger brain activations for L1 versus L2 emotional words in sectors of the posteromedial cortex involved in attention modulation, episodic memory, and affective processing. While fMRI findings are consistent with the hypothesis of a stronger emotional resonance when processing words in a native language, our overall pattern of results points to the different sensitivity of behavioural and hemodynamic responses to emotional information in the two languages of bilingual speakers.

## Introduction

Goal-directed behaviour requires the ability to override internal impulses or the automatic attentional capture by stimuli that interfere with ongoing task performance (Diamond, 2013). Frequently, the ability of inhibitory control is applied in affectively charged contexts, where interference is produced by affectively salient stimuli on task-related processing (Inzlicht et al., 2015; Aïte et al., 2018). Although most of the work on emotional processing has been carried out with monolingual participants, the last decade has witnessed an upsurge of interest in the processing and control of emotional information in bilingual speakers (see, for reviews, Rosselli et al., 2017; Kazanas et al., 2019; Del Maschio et al., 2022). In this context, the main question is to determine whether the processing of emotional information has the same characteristics in the native language (L1) as in the second language (L2) of bilinguals. Indeed, it has been suggested that emotions are dulled when using one’s L2, possibly because non-native languages are typically learned and used in contexts that do not allow L2 semantics to be grounded on the emotional experiences that characterise the semantic representation of L1 (e.g., Pavlenko, 2012). However, research to date provides an inconsistent support to such claim, with some studies pointing to an attenuated emotionality in the L2 relative to the L1 (e.g., Harris et al., 2003; Dewaele, 2004; Winskel, 2013), others showing that emotional information is processed similarly in both languages (Ferré et al., 2010; Altarriba and Basnight-Brown, 2011; Ponari et al., 2015), and still others reporting a larger emotional effect in the L2 than in the L1 (Ayçiçeği-Dinn and Caldwell-Harris, 2009; Caldwell-Harris et al., 2011). These divergences may be attributed, at least in part, to the diversity of methods and tasks that have been used to investigate the processing of affective language in bilinguals (see, Kazanas et al., 2019). For example, whereas introspective studies assessed bilinguals’ perception of emotionality in their two languages (e.g., Dewaele, 2004, 2008), behavioural studies used a range of different paradigms – such as lexical decision (e.g., Ponari et al., 2015), word recall (e.g., Ferré et al., 2010), and affective priming (e.g., Degner et al., 2012) – to investigate the effects of the emotional content of words in L1 and L2. Furthermore, a number of psychophysiological and electrophysiological studies have been conducted to examine, respectively, the time course of emotional processing (e.g., Jończyk et al., 2016) and physiological markers of arousal (e.g., skin conductance, pupillary, and grip force responses –Eilola and Havelka, 2011; Toivo and Scheepers, 2019; Thoma et al., 2023) when reading or hearing emotional words presented in L1 and L2. In addition to methodological differences, the inconsistencies of previous findings may be due to speaker-related variables that have been shown to modulate emotionality effects across languages. For instance, the age of acquisition (AoA) of the L2 (e.g., Harris et al., 2003; Colbeck and Bowers, 2012), the context of learning of the two languages (e.g., Brase and Mani, 2017; Ferré et al., 2018), and the asymmetries of proficiency or use of a language over the other (e.g., Degner et al., 2012; Winskel, 2013) have been recognised as relevant factors in modulating the direction of the emotional effects. Typically, the emotional resonance of the L2 is reduced compared to that of the L1 in unbalanced bilinguals with a low level of L2 proficiency, who were born and raised in environments in which their L1 was dominantly spoken, and who learned their L2 late and/or in instructional settings. In this picture, a special relevance to the context in which languages are learned and habitually used is attributed by the “emotional contexts of learning hypothesis” (Harris et al., 2006), which predicts that learning a language in environments that are rich in emotional experiences leads to a stronger emotional resonance when processing information in that language. According to this hypothesis, L1 has a stronger emotional resonance because it is typically acquired in a family context that carries the full range of human emotions, whereas an L2 would feel less emotional when learned in formal contexts (e.g., classroom) that do not provide extensive opportunities for the integration of the L2 lexicon with emotional experiences (see, Caldwell-Harris, 2014).

The aim of the current work is to shed additional explanatory light on the mechanisms underlying emotional processing in the bilinguals’ two languages. While behavioural evidence has steadily increased in the last few years, the number of studies that paired behavioural tasks with neuroimaging methods to investigate emotion processing in bilinguals remain surprisingly scant (see, Chen et al., 2015; Hsu et al., 2015; Sulpizio et al., 2019). Here, we used an emotional interference paradigm (the Emotional Stroop Task – EST) and functional magnetic resonance imaging (fMRI) to provide a neurocognitive characterisation of emotional interference in a group of native Italian speakers who learned English as an L2.

In the most popular emotion-word version of the EST (Gotlib and McCann, 1984; Williams et al., 1996), participants need to suppress interference from distracting emotional information (i.e., the emotional content of a word) in order to maintain ongoing task demands (e.g., naming or categorising the ink colour in which the word is presented). The characteristic finding from the EST is an interference effect – referred to as the “Emotional Stroop effect” – reflected in longer response times (RTs) to emotionally valenced words (e.g., “death”) relative to neutral words (e.g., “closet”). Although the specific mechanisms underlying this effect remain a matter of debate (see, Algom et al., 2004), a general difference in processing emotional versus neutral words has been interpreted as suggestive of a fast and implicit attentional capture by emotional words (e.g., Williams et al., 1996), presumably due to the motivational and adaptive significance of affective stimuli (Lang et al., 1990). At the neural level, performing interference tasks in affectively charged contexts has been reported to engage regions commonly associated with cognitively controlled processes, such as the dorsolateral prefrontal cortex (DLPFC) and the anterior cingulate cortex (ACC), but also fronto-limbic and fronto-insular circuitries implicated in the processing and regulation of affective stimuli (e.g., Cromheeke and Mueller, 2014; Hung et al., 2018). Fronto-limbic structures are also engaged during EST execution in monolinguals, although stimulus characteristics (e.g., positive vs. negative valence), task versions (e.g., the classic “colour-word” version vs. the “word-face” variant), and task demands (e.g., high vs. low emotional conflict) may reflect in specific patterns of brain activity (Song et al., 2017).

In bilinguals, prior behavioural evidence from the EST is mixed, with studies pointing either to a reduced emotional resonance of the L2 relative to the L1 (e.g., Eilola and Havelka, 2011; Winskel, 2013), or to the same amount of emotional interference in the bilinguals’ two languages (e.g., Eilola et al., 2007; Grabovac and Pléh, 2014). The variable characteristics of the tested samples seem relevant in explaining these divergences. In particular, the automaticity of emotional processing across languages does not seem to differ significantly in bilinguals who are highly proficient in both their languages and/or immersed in bilingual environments (e.g., Eilola et al., 2007; Sutton et al., 2007; Grabovac and Pléh, 2014). Conversely, in at least one study (Winskel, 2013), late unbalanced bilinguals who were less proficient in their L2 than their L1 showed less automatic activation of emotion words in their L2 (i.e., the Emotional Stroop effect was restricted to L1).

To the best of our knowledge, the current study represents the first attempt to examine, with behavioural and fMRI data, emotionality effects on bilinguals’ word processing during EST execution. While some previous neuroimaging work has shown increased activity in a network of cortico-limbic structures when processing emotional information in L1 compared to L2 (Hsu et al., 2015), other studies reported less cohesive findings, with region-specific differences in activation for emotional vs. neutral words between languages (Chen et al., 2015). It is worth noting, however, that these previous findings rely on data collected with tasks other than the EST, and by sampling participants whose language experience greatly differed across their two languages. We investigated mechanisms of emotional control in a sample of native Italian speakers who were first exposed to English during childhood, mainly in instructional settings rather than immersion, and who were proficient in their L2 at time of testing. Therefore, we predicted that the amount of emotional interference would not significantly differ across our bilinguals’ languages, mainly as a function of the proficiency attained in their L2. This would reflect, at the behavioural level, in a similar pattern of word processing in both languages, with longer RTs for emotional versus neutral words, but no significant interaction between word type and language. At the neural level, we expected to find some general differences in activation as a function of word type in frontal-subcortical networks typically engaged in the processing and control of emotional information.

## Materials and methods

### Participants

Thirty-six (*N* = 36) young adults volunteered to participate in the study (22 F; M_age_ = 24.38 ± 4.21 years; M_years of education_ = 16.85 ± 1.80). Participants were recruited via advertisements on university bulletin boards and social media. All participants were right-handed as determined by the Edinburgh Handedness Inventory (Oldfield, 1971) (Edinburgh score = 0.89 ± 0.14 points). All were native Italian speakers who learned English as an L2 and had normal or corrected-to-normal visual acuity. No participant had a history of neurological or psychiatric disease or substance abuse, nor was in treatment with psychiatric medications. Due to task requirements, participants were screened for colour blindness before the MRI scanning session. The study was approved by the Human Research Ethics Committee of the San Raffaele Hospital (Milan, Italy). Informed consent was obtained from all participants.

Participants’ bilingual language background was assessed through the Language History Questionnaire (version 3) (LHQ3) (Li et al., 2020). For each participant, L2 AoA, L2 self-reported proficiency, and language dominance were collected. L2 AoA was operationalized as the lowest age at which participants began to speak, read, write, or listen to in the L2. L2 self-reported proficiency was calculated as the weighted sum of participants’ self-rated proficiency on different components of L2 knowledge (i.e., listening, speaking, reading, and writing). Language dominance was determined, for both L1 and L2, as an aggregate score of self-reported proficiency in a language and the estimated time spent every day using that language in different activities (e.g., listening to podcasts and reading). L2–L1 dominance was computed as the ratio of the dominance score of the L2 against that of the L1. The ratio score ranges from 0 to 1, and indicates to what extent a participant is exposed to both languages (0 = the participant is exposed only to the L1; 1 = the participant is equally exposed to L1 and L2). For a detailed description of the LHQ3 measures and their calculation, see Li et al. (2020). Participants’ objective proficiency in the L2 was assessed through the English Proficiency Test (EPT). The EPT (developed by Transparent Language),^1^ includes 40 multiple-choice items. Thirty questions evaluate English grammar and conversational knowledge (e.g., participants had to fill in the blank spaces within a sentence with the correct option), and 10 questions assess text comprehension abilities (i.e., participants had to correctly answer questions regarding short English texts). Based on the scores obtained at the EPT (*M* = 35.25 points; SD = 3.59; range: 25–40), participants were classified as medium-to-highly proficient. Twenty-one participants (58.33%) learned English exclusively in instructional settings; 4 participants (11.11%) learned English exclusively in immersive contexts; 11 participants (30.55%) learned English in mixed contexts (i.e., classroom + self-learning / classroom + immersion / classroom + self-learning + immersion). The descriptive statistics of bilinguals’ background measures are reported in Table 1.

**TABLE 1:** Participants’ bilingual language background.

	Mean ± SD (range)
L2 AoA	5.69 ± 2.90 (0–11)
L1 self-reported proficiency	0.99 ± 0.02 (0.89–1)
L2 self-reported proficiency	0.74 ± 0.14 (0.43–1)
L2–L1 dominance ratio	0.65 ± 0.15 (0.40–0.99)
L2 objective proficiency	35.34 ± 3.60 (25–40)

Mean, standard deviation (SD) and range are reported for L2 age of acquisition (AoA), L2 self-reported proficiency, L2-to-L1 dominance ratio, and L2 objective proficiency (i.e., grammar and conversational knowledge). The scores for L2 self-reported proficiency and L2-to-L1 dominance ratio range from 0 to 1; the score for the L2 objective proficiency ranges from 0 to 40. L2, second language; L1, native language.

### Stimuli

Italian (L1) words were selected from the Italian adaptation (Montefinese et al., 2014) of the Affective Norms for English Words database (Bradley and Lang, 1999). English (L2) words were selected from the Glasgow Norms database (Scott et al., 2019). Each word was originally rated on a 9-point Likert scale across 6 psycholinguistic dimensions (for details, see Montefinese et al., 2014; Scott et al., 2019). For each language, we selected 2 sets of 50 words, one emotional (i.e., negatively valenced) and one neutral. The final set of stimuli included 200 words. Within each language, emotional and neutral words were comparable in terms of several psycholinguistic variables, but differed in terms of valence and arousal (all *p*s < 0.001). Across languages, the valence and arousal scores were comparable for both emotional and neutral words (all *p*s > 0.1) (see Table 2). Crucially, the stimuli were matched across L1 and L2 in terms of their affective dimensions [i.e., L1 (EWs) vs. L2 (EWs) valence: *p* = 0.161; L1 (NWs) vs. L2 (NWs) valence: *p* = 0.562; L1 (EWs) vs. L2 (EWs) arousal: *p* = 0.123; L1 (NWs) vs. L2 (NWs) arousal: *p* = 0.171].

**TABLE 2:** Psycholinguistic properties of the stimuli used in the Emotional Stroop Task (EST).

	L1			L2		
	EWs	NWs	*p*	EWs	NWs	*p*
Frequency	39.20	64.39	>0.3	44.39	45.92	>0.9
OLD	1.55	1.54	>0.9	1.81	1.80	>0.9
Number of letters	7.04 (1.74)	7.14 (1.95)	>0.7	6.26 (2.08)	6.26 (2.10)	>0.9
Concreteness	5.63 (1.26)	6.02 (1.87)	>0.2	3.87 (1.17)	4.29 (1.47)	>0.1
Imageability	6.63 (0.88)	6.68 (1.46)	>0.8	4.47 (0.95)	4.54 (1.49)	>0.7
Valence	2.21 (0.52)	5.04 (0.27)	<0.001	2.06 (0.53)	5.13 (0.27)	<0.001
Arousal	6.20 (0.81)	5.03 (0.51)	<0.001	5.98 (0.60)	4.80 (1.18)	<0.001

The frequency values for the L1 (Italian) and the L2 (English) were taken, respectively, from SUBTLEX_IT (https://osf.io/zg7sc/) and SUBTLEX_UK (https://osf.io/zq49t/). For each word, the mean Orthographic Levenshtein Distance (OLD) was calculated using the vwr library (https://cran.r-project.org/src/contrib/Archive/vwr/) running on R, version 4.1.2. Concreteness, Imageability, Valence, and Arousal scores were taken from the Italian adaptation (Montefinese et al., 2014) of the Affective Norms for English Words (Bradley and Lang, 1999) and the Glasgow norms database (Scott et al., 2019). EWs, emotional words; NWs, neutral words.

### Design and procedure

The study employed a 2 × 2 design with Language (words presented in Italian vs. English) and Word Type (negative and neutral words) as within-subjects factors. An event-related design was used to administer the EST in the MRI scanner. The experimental session consisted of 2 runs, 1 run per language, each comprising 2 blocks of 100 trials: 50 emotional trials and 50 neutral trials (2 runs, 4 blocks and 400 trials in total). Words were printed in capital letters in 1 of 4 colours: red (RGB: 255, 0, 0), blue (RGB: 1, 50, 187), yellow (RGB: 255, 255, 0), and green (RGB: 0, 255, 0). Each word was presented twice in each language (i.e., once per block), and each of the 4 colours was presented 25 times within each block. The two fMRI runs were interleaved with the acquisition of a T1 weighted MR image, and their presentation order was counterbalanced across participants. Within each block, trials were arranged in a fixed pseudo-randomised order, so that no more than 4 consecutive trials belonging to the same condition or having the same word colour were presented to participants.

Each trial started with the presentation of a fixation cross (350 ms) appearing in the centre of the screen, and indicating where the subsequent stimulus would appear. In each trial, a word was presented centrally on a black background for 2,000 ms, irrespective of whether the participant had made a response or not. The inter-trial interval (ITI) was jittered with the Dale’s exponential function (Dale, 1999) (mean = 1,720.86 ms; range = 1,127–3,259 ms). Each run lasted approximately 15 min (∼8 min per block). The total scanning time for each participant (including the acquisition of structural data) was approximately 45 min.

Before completing the EST, participants read standardised instructions and underwent a training session inside the scanner. They were instructed to indicate, as quickly and accurately as possible, the ink colour of the words presented to them, without paying attention to the word content. The training session comprised 56 trials (28 trials – 14 emotional, 14 neutral – for each language condition). The stimuli used in the training session were not employed in the experimental session. Participants’ responses were given by pressing a button on an MRI-compatible four-button response box. The Presentation software^2^ was used to present stimuli and collect responses. Accuracy and response latencies in milliseconds (ms) were recorded.

As a complement of the experimental task, at the end of the fMRI session outside the scanner, affective ratings were collected for the emotional and neutral words used as stimuli in the EST. In particular, participants were asked to evaluate the words on two 7-point scales in terms of Valence (from 1 = fully unpleasant to 7 = fully pleasant) and Arousal (from 1 = completely calm/relaxed to 7 = totally activated). Within each language condition (L1 and L2) words were presented in a randomised order, while the order of the language blocks matched that of the fMRI session. Participants were also asked to indicate whether they knew or not the meaning of each L2 word.

### MRI acquisition

Magnetic resonance images were acquired with a 3 Tesla Philips Ingenia CX MR system (Philips HealthCare, Best, Netherlands) equipped with a 32 channels SENSE head coil at C.E.R.M.A.C. (Centro Eccellenza di Risonanza Magnetica ad Alto Campo) of San Raffaele Hospital in Milan (Italy).

For all participants, a high-resolution T1-weighted MPRAGE (Magnetisation Prepared Rapid Gradient Echo) structural image was acquired with the following parameters: Repetition Time (TR) = 9.9 ms, Echo Time (TE) = 4.9 ms, L2ip Angle = 8°, Field of View (FOV) = 260 mm, matrix size = 256 × 256, number of slices = 243, slice thickness = 1.4 mm, voxel size = 0.7 mm × 0.7 mm × 0.7 mm isotropic; Phase Encoding Direction (PE) = R/L; whole brain coverage.

Functional scans were acquired with a fast speed Echo Planar Imaging (EPI) sequence [TE = 33 ms; TR = 2,000 ms; L2ip Angle (FA) = 85°; number of volumes per run = 218; FOV = 240 × 240 mm; matrix size = 80 × 80; 35 axial slices per volume; slice thickness = 3 mm; interslice gap = 0.75; voxel size = 3 mm × 3 mm × 3 mm; PE = A/P; SENSE factor = 2; whole brain coverage]. Four dummy scans preceded each run to optimise EPI image signal.

### Pre-processing

Structural and functional data were pre-processed using SPM12,^3^ running on Matlab 2013b. First, the origin of each T1w image was manually set to match the Anterior Commissure – Posterior Commissure (AC-PC) line. Then, T1w images were bias-corrected for intensity inhomogeneities and segmented using the “unified segmentation and normalisation” function in SPM12 (Ashburner and Friston, 2005). Finally, non-cerebral tissue was removed from bias-corrected structural T1w images by means of the “Image Calculator” SPM function (i.e., skull stripping). Functional volumes were slice-time corrected using the first slice as reference point, then realigned to the first volume and unwarped to correct for motion artefacts and geometric distortions. Realigned functional volumes were coregistered to the bias-corrected skull-stripped structural image and normalised to the standard Montréal Neurological Institute (MNI) template. After normalisation, functional volumes were resampled to 2 mm × 2 mm × 2 mm voxels and smoothed with a 10 mm full width at half-maximum (FWHM) Gaussian kernel. For each participant, functional volumes were checked for excessive head motion (>2 mm).

### Statistical analyses

#### Behavioural analyses

Behavioural analyses were performed using R software (version 4.1.2) (R Development Core Team, 2015). The effects of Word Type (i.e., emotional vs. neutral words) and Language (i.e., L2 vs. L1) on RTs, accuracy, valence, and arousal ratings^4^ were tested with mixed-effects models. Participants and items were modelled as random intercepts. Word Type and Language were entered as fixed effects into the model, and each effect was tested for its significance by comparing a model which included the fixed term of interest against a model in which it was not present (i.e., likelihood ratio tests). Fixed effects were retained when they increased the goodness of fit. In case of significant interactions, all the lower-order terms involved were retained. *Post hoc* comparisons were performed using the “emmeans” package (Lenth, 2021).

Reaction times were analysed by means of a linear mixed-effects model with raw RTs as dependent variable and Word Type and Language as predictors. Response accuracy was analysed by means of a mixed-effects logistic regression model run with correctness of response as dependent variable. Both models were implemented using the “lmerTest” package (Kuznetsova et al., 2017).

Valence and arousal ratings were analysed by means of mixed-effects ordinal logistic regression models run with valence or arousal as dependent variables. These models were implemented with the “ordinal” package (Christensen, 2019).

#### fMRI analyses

A two-level summary statistic approach was implemented in SPM12 to analyse functional data. Three participants were excluded from functional analyses for excessive head motion (>3 mm). Thus, functional analyses were performed on a sample of 33 participants.

##### First level analysis

Evoked responses for the experimental conditions were entered into a General Linear Model (GLM) and modelled with the canonical haemodynamic response function (HRF). The onset times of the trials were specified for each of the four blocks (i.e., Neutral L1, Emotional L1, Neutral L2, and Emotional L2). Realignment parameters were entered as nuisance covariates. Student’s *t*-test linear contrasts were computed for each participant. Main effects of Word type (i.e., Emotional and Neutral) and Language (i.e., L2 and L1) were estimated along with the following contrasts: Word Type (Emotional > Neutral, Emotional < Neutral), Language (L2 > L1, L2 < L1), and their interactions (Neutral L2 > Emotional L2, Neutral L2 < Emotional L2; Neutral L1 > Emotional L1, Neutral L1 < Emotional L1; Emotional L1 > Emotional L2, Emotional L1 < Emotional L2; Neutral L1 > Neutral L2, Neutral L2 > Neutral L1). Temporal autocorrelation was accounted for with an AR (1) regression algorithm imposing a high-pass filter of 128 s, which removed slow signal chains with a longer period.

##### Second level analysis

The contrast images obtained at the single-subject level were entered into a one sample *t*-test model to determine their significance at the group level. A voxel-wise whole brain analysis was performed. Statistical threshold was set at *p* < 0.001 at the voxel level, and *p*-family-wise error (FWE) corrected < 0.05 at the cluster level.

## Results

### Behavioural results

Descriptive statistics of behavioural variables are reported in Table 3. RTs smaller than 300 ms and greater than 2,000 ms were treated as outliers (e.g., Franken et al., 2009). No trials were removed from subsequent analyses according to this criterion. Response accuracy was consistently high in all conditions and was thus not subjected to further analysis.

**TABLE 3:** Descriptive statistics of behavioural data and affective ratings.

	L2 emotional	L2 neutral	L1 emotional	L1 neutral
Accuracy	98.11 ± 1.65	97.70 ± 2.06	98.33 ± 1.53	97.64 ± 1.99
RTs	706.59 ± 250.99	708.61 ± 242.16	701.75 ± 227.21	725.77 ± 252.45
Valence	2.54 ± 1.60	4.09 ± 1.17	2.29 ± 1.22	3.94 ± 0.99
Arousal	4.69 ± 1.82	2.04 ± 1.43	4.59 ± 1.77	1.99 ± 1.41

Mean and standard deviation (SD) for accuracy (% correct responses), reaction times (RTs) (in ms), and valence and arousal ratings (7-point scale) in the Emotional Stroop Task by Word Type (emotional and neutral) and Language (L1 and L2).

**FIGURE 1:**
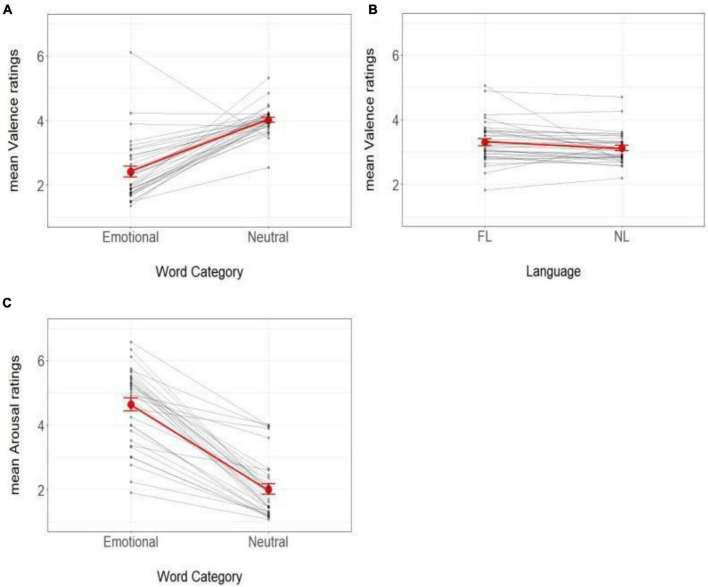
Effects of word category language on participants’ affective ratings. Valence and arousal ratings (on a 7-point scale) as a function of Word Type (emotional and neutral) and Language (L1 and L2). **(A)** Mean word valence as a function of Word Type. **(B)** Mean word valence as a function of Language. **(C)** Mean word arousal as a function of Word Type. Black dots represent participants’ individual ratings in each condition. Grey lines connect different observations from the same participant. Red dots, connected through red lines, represent the mean rating scores in the whole sample. Error bars represent standard errors. FL, Foreign Language (L2); NL, Native Language (L1).

In the RTs analysis, no effect reached significance (all *t*s < 1.4, all *p*s > 0.1). In the analyses of affective ratings, the analysis of valence showed a significant effect of Word Type (Likelihood Ratio Test [LRT] = 375.47, *p* < 0.001, *b* = −1.52, SE = 0.04, *z* = −31.03) – indicating that emotional (negative) words were perceived as less pleasant than neutral words – and a significant effect of Language (LRT = 5.97, *p* = 0.01, *b* = 0.11, SE = 0.04, *z* = 2.46) – indicating that L1 words were perceived as less pleasant than L2 words. The interaction between Word Type and Language was not significant (LRT = 0.05, *p* > 0.8). The analysis of arousal showed a significant main effect of Word Type (LRT = 327.06, *p* < 0.001, *b* = 1.98, SE = 0.07, *z* = 27.38), with emotional (negative) words being perceived as more arousing than neutral words. No other effect reached significance (all *ps* > 0.1). The patterns of Valence and Arousal ratings are presented in Figure 1.

### fMRI results

**FIGURE 2:**
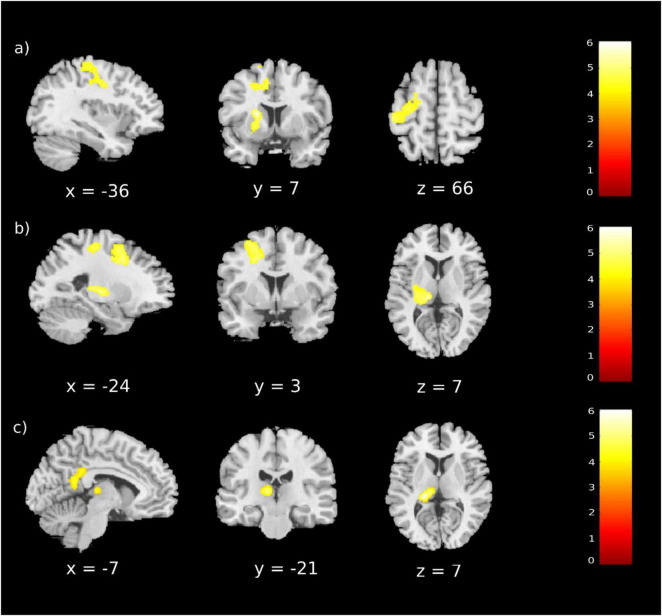
Brain activations for significant contrasts. Brain activity elicited by specific conditions at the group-level (voxel-level: *p* < 0.001 uncorrected; cluster-level: *p*-FEW < 0.05). **(a)** Neutral words > Emotional words; **(b)** L2 Neutral words > L2 Emotional words; **(c)** L1 Emotional words > L2 Emotional words. No other contrast led to significant effects. Coordinates (*x*, *y*, *z*) are reported in MNI space. Colour bar represents *t*-values.

Functional magnetic resonance imaging results are reported in Table 4 and Figure 2. A significant main effect of Word Type was observed, revealing that, regardless of language condition, neutral (vs. emotional) words elicited a stronger left-lateralised activity in the putamen, thalamus, and sensorimotor cortex (see Table 4 and Figure 2a). No significant main effect of Language was observed (*p*-FWE > 0.05). Significant interactions between Word Type and Language also emerged. In particular, the processing of neutral (vs. emotional) words in the L2 condition elicited a greater left-lateralised activation in the superior frontal cortex, in the sensorimotor cortex, and in the thalamus (see Table 4 and Figure 2b). Moreover, the processing of L1 (vs. L2) emotional words elicited a greater activation in the left thalamus, in the right posterior cingulate cortex, as well as mesial and lateral aspects of the left parietal cortex (see Table 4 and Figure 2c). No further contrast reached significance (*p*-FWE > 0.05).

**TABLE 4:** Contrast analyses.

Contrast	*p*-FWE	*k*	*t*-value	Hem	Labels (AAL)	MNI coordinates		
						*x*	*y*	*z*
Neutral words > emotional words	0.007	697	4.98	L	Putamen	−20	8	14
			4.20	L	Putamen	−26	−14	2
			4.15	L	Thalamus	−22	20	−2
	<0.0001	1,276	4.48	L	Postcentral gyrus	−44	−10	44
			4.13	L	Precentral gyrus	−28	−16	60
			4.09	L	Middle cingulum	−14	6	44
L2 neutral words > L2 emotional words	<0.0001	2,041	5.09	L	Superior frontal cortex	−20	8	44
			4.42	L	Precentral gyrus	−30	−20	56
			4.23	L	Postcentral gyrus	−38	−32	56
	0.009	620	4.56	L	Thalamus	−12	−24	8
			4.49	L	Thalamus	−20	−14	4
			4.23	L	Thalamus	−24	−26	8
L1 emotional words > L2 emotional words	0.043	317	5.65	L	Thalamus	−20	−30	8
			4.78	L	Thalamus	−12	−24	8
			3.55	L	Thalamus	−12	−16	4
	<0.0001	1,276	4.66	R	Posterior cingulate cortex	2	−36	32
			4.65	L	Precuneus	−16	−54	40
			4.55	L	Angular gyrus	−22	−50	36

Contrasts leading to significant effects (voxel-level: *p* < 0.001 uncorrected; cluster-level: *p*-FEW < 0.05). Coordinates (*x*, *y*, *z*) are reported in MNI space. Region labels are based on the Harvard-Oxford Atlas. *k*, number of voxels within each significant cluster; Hem, hemispheric lateralisation.

## Discussion

The effects of emotional information across a bilingual’s two languages, as well as the modulatory role of bilinguals’ experience on the direction of such effects, are still a contentious issue with no clear-cut answers. We aimed to shed light on bilinguals’ emotional processing in L1 and L2 during an emotional interference task (the EST), and to assess - with fMRI data - the corresponding neural correlates. Building on previous research, we expected that the amount of emotional interference would not significantly differ across our bilinguals’ languages, mainly as a function of the high level of L2 proficiency attained by our sample. In what follows, we start by discussing the behavioural results, then we move to the fMRI data, and we conclude by outlining the relevance of our findings for research on affective processing in bilingual speakers.

### Behavioural results

We observed a similar pattern of word processing in both our participants’ languages. This finding concurs, for example, with those of Sutton et al. (2007) and Grabovac and Pléh (2014), who presented emotional and neutral words to early proficient bilinguals, and that of Eilola et al. (2007), who presented emotional and neutral words to late proficient bilinguals. Sutton et al. (2007), in particular, presented negative and neutral words to early Spanish-English bilinguals who were dominant in their L2, and found significant effects of word type and language on word processing (i.e., bilinguals were slower in categorising the colour of negative words and faster in categorising the colour of L2 words) but no significant interaction between the two factors (i.e., emotional words produced interference in both languages). Eilola et al. (2007) used positive, negative, taboo, and neutral words in late Finnish dominant bilinguals who reached a high level of proficiency in their L2 (English). Although significant differences in RTs between negative and neutral words, as well as taboo and neutral words were observed, language did not produce a main effect, nor interacted with word type on colour categorisation. A similar pattern of results (a word type effect, with no language differences or interaction effects) was observed in a Hungarian minority group assimilated into a Serbian majority culture who acquired their L2 early and reached high levels of proficiency in that language (Grabovac and Pléh, 2014). At first glance, when interpreted alongside this earlier evidence, the lack of a reduced Emotional Stroop effect in L2 versus L1 in our study may be due to the high level of proficiency attained by our participants in their second language. It is worth noting, however, that although the above mentioned studies did not report a significant interaction between language and word type on colour categorisation, they did report an Emotional Stroop effect of comparable size in L1 and L2. By contrast, we failed to detect any significant difference in responding to negative and neutral words in both our participants’ languages. As suggested by Liao and Ni (2022), who focused on the processing of L2 words in Chinese-English bilinguals and failed to report an Emotional Stroop effect on RTs, null effects of emotionality may be attributed to the shallower level of processing at which the EST operates relative to more explicit tasks. On similar lines, Crossfield and Damian (2021) matched a set of neutral, negative, and positive words on multiple psycholinguistic variables other than valence, and used them both in the EST and in a Lexical Decision Task (LDT) administered to monolingual participants. Results indicated a significant processing advantage for positive words over negative and neutral words in the LDT, whereas valence alone did not produce any significant effects in the EST. It was concluded that significant effects of valence would be constrained to tasks where this variable is relevant for task success, and/or tasks that require a deeper level of processing. The results from the affective rating task we administered to participants after EST completion are compatible with this suggestion. We found, in particular, that negative words were perceived as less pleasant and more arousing than neutral words, and that L1 words were overall perceived as less pleasant than L2 words. Whereas in the EST participants are instructed to ignore the words’ content when categorising their responses, affective ratings are expected to drive, to a larger extent, the allocation of top-down attentional resources to the connotative meaning of the stimuli. The different mechanisms underlying word processing in implicit (i.e., EST) and explicit tasks (i.e., affective rating) may account for the task-specific effects of emotional information reported here and in previous research on emotional interference or attentional bias toward negative content. As a side note, it is worth mentioning that we used a version of the EST that required manual responses (i.e., key presses). The Stroop literature has shown that response modality modulates the magnitude of Stroop interference, which is typically smaller in task versions that require manual (vs. verbal) responses. This is arguably because the interference observed with verbal responses results from the contribution of task, semantic, and response conflicts, whereas task conflict does not significantly contribute to the interference observed with manual responses (see, e.g., Kinoshita et al., 2018; Augustinova et al., 2019; Scaltritti et al., 2022).

### fMRI results

Among the numerous studies that used the EST to probe emotional interference processes in both healthy and clinical populations, many reported altered psychophysiological and neural (re)activity to negative stimuli in the absence of any detectable behavioural effect (e.g., Compton et al., 2003; Thomas et al., 2007; Fan et al., 2016). The overall pattern of findings reported in this study is in line with such previous evidence, possibly due to the different sensitivity of behavioural and hemodynamic responses to the emotional content of words in L1 and L2 during EST execution. Building on previous research, we expected to find some general differences in activation as a function of word type in frontal-subcortical networks typically engaged in emotional control, but no (or only marginally) significant interactions between word type and language. Contrary to our predictions, the key fMRI finding reported in this study is the significant interaction between word type and language – with stronger activations for L1 versus L2 emotional words – in sectors of the posteromedial cortex involved in attention modulation, episodic memory, and emotional processing. The posteromedial cortex receives major inputs from the dorsal visual stream and somatosensory areas, as well as from regions with functions related to emotion and social behaviour, including the subgenual ACC and the orbitofrontal cortex (see, Rolls, 2019). Moreover, the ventral aspects of the posterior cingulate cortex (PCC) and the adjacent retrosplenial cortex have reciprocal connections with memory structures in the medial temporal lobe (Vogt et al., 2001; Leech and Sharp, 2014). Consistent with its anatomy and connectivity, the PCC, in particular, has been found to be engaged by a range of tasks that involve the modulation of attentional focus (e.g., Hahn et al., 2007; Pearson et al., 2011), episodic memory (see, Rugg and Vilberg, 2013), the retrieval of emotionally salient contextual information (e.g., Maratos et al., 2001), and the evaluation of emotional versus neutral verbal stimuli (e.g., Maddock et al., 2003). The stronger activations for L1 versus L2 emotional words in relevant sectors of the posteromedial cortex is consistent with the hypothesis of a stronger emotional resonance when processing words in a native language. Noticeably, previous behavioural and psychophysiological evidence suggests that L2 proficiency – more than other variables – modulates the extent to which emotional resonance is dampened in a second language (e.g., Ferré et al., 2010; Caldwell-Harris et al., 2011; Degner et al., 2012; Champoux-Larsson and Nook, 2024). Whereas our behavioural data are compatible, at least in part, with this tenet, our fMRI findings seem to downplay the significance of proficiency in favour of other characteristics of bilinguals’ language background, such as learning context. The “emotional context of learning hypothesis” (Harris et al., 2006) suggests that learning a language in the absence of emotion-based communicative interactions leads to a reduced emotionality of that language due to a weaker connection with emotion regulation systems. Our participants were proficient in their L2, and displayed a relatively balanced use of the two languages at time of testing (i.e., they were equally exposed to both L1 and L2 more than they were exposed to the L1 only, as indexed by the L2–L1 dominance ratio metric). However, they learnt English mainly in instructional or mixed contexts (only ∼10% of participants learned English exclusively via immersion), where the opportunities for affective linguistic conditioning are fewer compared to the contexts of acquisition of L1 (e.g., Pavlenko, 2008; Caldwell-Harris, 2014). Therefore, the increased affective response to negative content experienced in L1 (vs. L2) may be attributed to the stronger emotional resonances associated with that language, acquired in a context that was rich in emotional experiences.

Another aspect worth mentioning is that we failed to observe any significant effect of word type or interaction between word type and language in the amygdala, a key region in the brain circuitry of emotion (LeDoux, 2000). As suggested by Chen et al. (2015), however, the activation of the amygdala has been found more frequently in studies where the evaluation of the emotional content of words was explicitly required by the task at hand, and not when emotional valence was task-irrelevant. In addition, the neural systems associated with emotional reaction have been shown to be more active for emotional pictures than for words. This is arguably because pictures are perceived as more biologically salient and emotionally arousing than written verbal stimuli. In support of this hypothesis, a recent meta-analysis of fMRI data on implicit emotional processing in monolinguals (Feng et al., 2021) suggested that affective pictures and words modulate implicit emotional processing differently, and recruit distinct neural systems. In particular, only negative pictures, and not words, could reliably elicit activation in the amygdala.

Several issues of the present study call for caution when interpreting the results. First, as we did not manipulate dimensions of bilinguals’ language background such as L2 proficiency or learning context, inferences about the contribution of these variables on emotional processing are only tentative. Second, although this is one of the very few studies that paired a behavioural task with fMRI to investigate affective processing in bilinguals, the evidence we provide comes from the investigation of single, decontextualized affective stimuli. The implementation of natural language in more ecological paradigms (e.g., naturalistic viewing paradigms –Sonkusare et al., 2019; Bellini et al., 2024) may help future research drawing a more reliable and lifelike picture of how bilinguals process affective language in everyday communication.

Overall, a number of theoretical and methodological implications can be drawn from our findings. A first theoretical implication is that the role of bilinguals’ languages in their affective repertoires is complex, and arguably conditional upon numerous factors. In our sample – which comprised proficient bilinguals who learned their L2 mostly in instructional or mixed contexts – stronger activations were observed for processing L1 versus L2 emotional words in sectors of the posteromedial cortex involved in attention, memory retrieval, and affective processing. However, the interpretation of fMRI findings could not be guided by our behavioural results, which showed no difference in the automaticity of emotional word processing across L1 and L2. This is possibly due to the different sensitivity of behavioural and hemodynamic responses to the emotional content of words in L1 and L2, but may also suggest that the EST is not suitable to capture potential emotionality effects on word processing across languages. The EST has been widely deployed in clinical studies to investigate individual differences in emotional processing by using emotional words related to a particular individual’s pathology or dysfunctional personality trait (e.g., anxiety, phobia, depression, substance addiction – see, Williams et al., 1996). However, when applied to explore implicit affective word processing in healthy speakers – both bilinguals and monolinguals – this task has produced mixed results, often in contrast with psychophysiological and brain imaging data (see, for reviews, Phaf and Kan, 2007; Jończyk, 2016). The present study provides further evidence that the EST, at least in its colour-word “manual” version, may not be suitable to capture potential emotionality effects that can be seized through fMRI analysis. Future research should possibly make use of other tasks involving emotional interference, or other versions of the EST (e.g., the “word-face” variant) involving more intense emotional conflict (see, Song et al., 2017).

## Conclusion

We examined, with behavioural and fMRI data, emotionality effects on word processing in a group of proficient bilinguals during an emotional interference task (i.e., the EST). In spite of no detectable behavioural effects, we observed stronger brain activations for L1 versus L2 emotional words in sectors of the posteromedial cortex involved in attention, memory retrieval, and affective processing. This finding is consistent with the hypothesis that emotional resonance is stronger when processing words in a native language. As the EST apparently fails to capture effects that can be seized through fMRI analysis, future studies should possibly make use of tasks more suitable for investigating emotional control processes in a bilingual’s two languages.

## Data availability statement

The raw data supporting the conclusions of this article will be made available by the authors, without undue reservation.

## Ethics statement

The studies involving humans were approved by the Human Research Ethics Committee of the San Raffaele Hospital (Milan, Italy). The studies were conducted in accordance with the local legislation and institutional requirements. The participants provided their written informed consent to participate in this study.

## Author contributions

ND: Conceptualisation, Investigation, Supervision, Writing – original draft, Writing – review & editing. SS: Conceptualisation, Supervision, Writing – review & editing. CB: Data curation, Formal analysis, Methodology, Writing – review & editing. GD: Data curation, Formal analysis, Writing – review & editing. MG: Data curation, Writing – review & editing. DB: Data curation, Formal analysis, Writing – review & editing. DF: Data curation, Methodology, Writing – review & editing. DP: Writing – review & editing. JA: Supervision, Writing – review & editing.

## Footnotes

1
https://www.transparent.com/

2
http://www.neurobs.com

3
https://www.fil.ion.ucl.ac.uk/spm/software/spm12/

4 Affective ratings were collected from 34 out of 36 participants (two participants were not able to complete the questionnaire).
